# Momelotinib – a promising advancement in the management of myelofibrosis in adults with anemia

**DOI:** 10.3389/fonc.2024.1411972

**Published:** 2024-06-25

**Authors:** Muhammad Furqan, Malik O. Oduoye

**Affiliations:** ^1^ Department of Medicine, King Edward Medical University, Lahore, Pakistan; ^2^ Department of Research and Education, Medical Research Circle, Goma, Democratic Republic of Congo

**Keywords:** myelofibrosis, JAKi inhibitors, momelotinib, MOMENTUM trial, SIMPLIFY trial, anemia

## Abstract

Myelofibrosis (MF) is a rare BCR-ABL negative myeloproliferative neoplasm characterized by clonal proliferation of stem cells, with mutations in JAK2, CALR, or MPL genes. MF presents in primary and secondary forms, with common symptoms including splenomegaly, anemia, and thrombocytopenia. Diagnostic criteria involve bone marrow examination and mutation studies. Current treatments are limited, with allogeneic stem cell transplant as the only curative option. Recent FDA approval of Momelotinib (MMB) offers new promise for MF patients with anemia. MMB, a JAK1/2 and ACVR1 inhibitor, effectively reduces spleen size, improves hemoglobin levels, and decreases transfusion dependency. The MOMENTUM trial compared MMB to danazol in JAK inhibitor-treated MF patients with anemia, showing MMB’s superior symptom relief and transfusion independence rates. Additionally, the SIMPLIFY-1 and SIMPLIFY-2 trials evaluated MMB in JAK inhibitor-naïve and experienced patients, respectively, confirming MMB’s non-inferiority to ruxolitinib in spleen volume reduction and highlighting its benefits in transfusion requirements. MMB’s unique dual inhibition mechanism addresses anemia by suppressing hepcidin production, thus enhancing erythropoiesis. These trials collectively suggest MMB as an effective treatment for MF, improving quality of life and offering a survival advantage for patients with anemia. Despite challenges, such as trial design limitations and adverse events, MMB represents a significant advancement in MF management, providing a new therapeutic option for a previously underserved patient population.

## Introduction

Myelofibrosis (MF) is a rare disease with an annual incidence rate of about 0.5–1.5 cases per 100,000 individuals in the United States, although increased prevalence is observed among the Ashkenazi Jews (1). MF is a *breakpoint cluster region-Abelson (BCR-ABL)* negative myeloproliferative neoplasm (2). This condition primarily affects the myeloid cell lineage, resulting in reduced production of red blood cells leading to anemia and platelets leading to thrombocytopenia. It can be categorized into two forms: primary MF and secondary MF, which includes cases arising after polycythemia vera and essential thrombocythemia. Primary myelofibrosis (PMF) is a type of myeloproliferative neoplasm characterized by the clonal proliferation of stem cells, often accompanied by mutations in *Janus kinase 2 (JAK2)*, calreticulin (*CALR)*, or myeloproliferative leukemia (*MPL)* genes (3). Bone marrow examination, including cytogenetic and mutation studies, is used to diagnose PMF. While mutations in JAK2, CALR, or MPL are typically expected, they are not always required (3). Although, over 80% of PMF patients have specific driver mutations, including JAK2 V617F (50–60%), CALR exon 9 mutations (20–25%), and MPL exon 10 mutations (6–7%), 10–15% of PMF patients lack these common driver mutations, a condition known as triple-negative PMF (4). The International Consensus Classification distinguishes between “prefibrotic” and “overtly fibrotic” PMF, with the former potentially resembling essential thrombocythemia (ET) in its presentation (3). Furthermore, approximately 15% of individuals diagnosed with ET or polycythemia vera (PV) eventually acquire a myelofibrosis-like manifestation, known as secondary myelofibrosis (SMF), post-ET myelofibrosis, or post-PV myelofibrosis. These conditions share comparable treatment approaches and outcomes with PMF (5).

MF is associated with splenomegaly, severely low blood counts, anemia and thrombocytopenia, debilitating symptoms, and an ability to develop vascular complications and a blast phase (persistent elevation in peripheral blood or bone marrow blasts of 20% or more) (6). Anemia in MF is a complex condition resulting from factors such as displacement of erythropoietic tissue by fibrotic stroma, suboptimal environments in extramedullary sites, and splenomegaly-induced RBC sequestration. Abnormal cytokine expression and inflammation in the bone marrow further disrupt erythropoiesis. An element of anemia in MF can occasionally be attributed to the effects of treatment (7).

## Pathogenesis of MF

Most cases of this disease are attributed to the mutation in the *JAK2, MPL, and CALR* genes. The proteins produced by *JAK2* and *MPL* genes are an integral part of the Janus kinase/signal transducer and activator of transcription *(JAK-STAT)* pathway, essential for regulating the proliferation and differentiation of the megakaryocytes (8). The low level of these proteins induces the proliferation of cells, while the high level of these proteins leads to the differentiation of the cells (9).

*MPLW515K/L/A/R* and *S505N* mutations, along with *JAK2*, are believed to activate *JAK2-STAT*, leading to cytokine-independent myeloproliferation. Mutant *CALR* may induce myeloproliferation by binding to *MPL* in the endoplasmic reticulum. Phenotypic differences exist between *JAK2V617F* and *CALR* mutations, with distinct clinical characteristics. Other high risk molecular mutations, including *ASXL1, SRSF2*, *U2AF1*, and *EZH2* are associated with inferior survival (10). Mutations such as *IDH1/IDH2, TP53, DNMT3A*, and *LNK* are more frequent in blast phase *MPN* (11). The pathogenetic role of these mutations involves disruption of epigenetic, RNA splicing, or transcriptional regulation. The stromal changes in MF, particularly intense in MF compared to PV and ET, involve abnormal proliferation of fibroblasts, endothelial, and mesenchymal cells. The role of fibroblasts in bone marrow fibrosis is supported by their resemblance to hematopoietic stem cells and monocytes (11).

The disruption of the JAK-STAT pathway is the hallmark of MF. This ultimately results in abnormalities related to the proliferation of megakaryocytes, which leads to the failure of the hematopoietic transcription factor GATA-binding factor 1 expression and affects granulocytes (basophils, neutrophils, and eosinophils) (12). Consequently, there is an increased release of inflammatory cytokines (e.g., TGF-β), causing myelo-proliferation with increased activity of fibroblasts leading to bone marrow fibrosis and the development of extramedullary hematopoiesis (13). In addition to this pathway, there are other pathways involved in MF, some of which are potential targets for drug treatment. About two-thirds of PMF patients carry the *JAK2V617F* mutation, while one-quarter have *CALR* gene mutations, and 10% each have MPL mutations or a “triple-negative” status (12). Furthermore, approximately 80% of patients also have additional variants in myeloid genes. Almost all of the patients develop anemia along the course of the disease.

## Standard therapies for MF

Current therapies for myelofibrosis are only partially effective, with allogeneic stem cell transplant being the only curative option in transplant-eligible patients despite the advancements in the availability of novel therapeutic agents (14). There is also an emerging practice of using ruxolitinib before a stem cell transplant to decrease spleen size, which has shown promise in improving the outcomes of the transplantation process by supporting quicker bone marrow recovery (15). Management strategies for patients with MF who are not candidates for transplantation are tailored based on their specific symptoms and their severity. In cases where patients exhibit no significant symptoms i.e., who do not display significant anemia (hemoglobin < 10 g/dl), splenomegaly (palpable spleen size > 10 cm), leukocytosis (leukocyte count > 25 × 10^9^/l), or marked thrombocytosis (platelet count > 1000 × 10^9^/l), an observational approach is recommended, with regular monitoring to track the emergence of any symptoms (16). However, the available treatments for PMF typically include corticosteroids, androgens, erythropoietin-stimulating agents (ESAs), and immunomodulatory drugs for managing MF-related anemia; hydroxyurea for managing symptomatic splenomegaly; and JAK inhibitors (such as ruxolitinib, fedratinib, and pacritinib) for managing splenomegaly or other symptoms with anemia. For those patients who are experiencing anemia but do not have splenomegaly or systemic symptoms (like fever or weight loss), the preferred treatment approach is to manage the anemia through red blood cell (RBC) transfusions (17). Supportive treatments aimed at reducing the frequency of transfusions are utilized alongside transfusions. While there is no universally agreed-upon protocol, options such as danazol (18) or ESAs are generally considered suitable (19). If a patient’s anemia and the need for frequent transfusions are not effectively managed with danazol or ESAs, alternative treatments might be considered. These include the combination of lenalidomide with prednisone (20) or thalidomide with prednisone (21), which can offer some benefits. Managing anemia and the side effects of repeated blood transfusions, such as iron buildup in the body, is critical and is often addressed through effective iron chelation treatments. Iron chelation therapy, such as deferasirox, maybe a feasible treatment option for iron overload in MF patients, including those who develop iron overload due to anemia worsening during JAK inhibitor treatment, particularly with ruxolitinib; the ‘RUX-IOL’ study demonstrated the safety and efficacy of combining ruxolitinib and deferasirox in MF patients. In a significant number of MF patients, the combination of ruxolitinib and deferasirox resulted in effective iron chelation and erythroid responses, leading to improved clinical outcomes without unexpected side effects (22). In cases where the patient suffers from noticeable splenomegaly but does not have anemia, the cytoreductive treatment i.e., hydroxyurea, demonstrates effectiveness in approximately 40% of individuals, although with frequently transient outcomes (23). The treatment strategy becomes more nuanced when patients exhibit both symptomatic splenomegaly and anemia. The choice of treatment in such scenarios is influenced significantly by the patient’s platelet count. For patients with a platelet count of 50,000/microL or higher, ruxolitinib is typically recommended (24). Conversely, for those with a platelet count below 50,000/microL, pacritinib is advised as it is deemed more suitable due to its safety profile and effectiveness (25). These standard therapies for patients not eligible for stem cell transplants are summarized in **Table 1**.

**Table 1:** Current therapies and disease management practices for MF.

Agents	MOA	Dosage	Outcomes	Toxicity
i. Symptomatic anemia only				
Danazol	Synthetic androgen that acts as a parital agonist at andorgen receptor	200 mg twice daily, but the dose can be increased to 400 mg twice daily, as tolerated.	Improves anemia in one-third of patients with PMF	▪ Hepatotoxicity ▪ Headache ▪ Masculinization ▪ Hirsutism ▪ Acne ▪ Edema
ESAs i.e., epoetin alfa and darbepoetin alfa	Induces erythropoiesis by stimulating the division and differentiation of committed erythroid progenitor cells	▪ Epoetin alfa: Recombinant human epoetin 40,000 to 60,000 units/week subcutaneously ▪ Darbepoetin alfa: 150 to 300 mcg subcutaneously every other week	ESAs are more effective than placebo for achieving transfusion independence and raising the level of Hb.	▪ Exacerbation of hypertenison ▪ Thrombosis ▪ Progression to acute myeloid leukemia
Prednisone	Suppresses the immune system by reducing activity and volume of the lymphatic system; suppresses adrenal function at high doses; inhibition of glucose transport, phosphorylation, or induction of cell death in immature lymphocytes.	0.5–1 mg/kg daily with tapering to the minimum effective dose	Improves anemia in one-third of patients with PMF	▪ Hyperglycemia ▪ Cushingoid changes ▪ Infectious complications ▪ Psychiatric disturbances
Lenalidomide plus prednisone	Immunomodulatory drug; selectively inhibits secretion of proinflammatory cytokines; enhances cell-mediated immunity by stimulating proliferation of anti-CD3 stimulated T cells	Lenalidomide 10 mg/d, orally continuous dosing on a 28-day cycle. For the first month, prednisone (30 mg/d by mouth) was given and was decreased to 15 mg/d for the second month and to 15 mg every other day for the third month.	Improves anemia in one-quarter to one-third of patients and may also reduce spleen size	▪ Congenital anomalies ▪ Hematologic toxicity ▪ Thromboembolic events
Thalidomide plus prednisone	Immunomodulatory drug; increase in natural killer cells and increased levels of interleukin-2 and interferon gamma; suppression of angiogenesis, prevention of free-radical-mediated DNA damage	Thalidomide 50 mg/day orally plus prednisone (beginning at 0.5 mg/kg orally per day and tapering over a period of three months)	Improves anemia in one-quarter to one-third of patients.	▪ Drowsiness ▪ Constipation ▪ Fatigue ▪ Paresthesias ▪ Neutropenia ▪ Thrombosis
ii. Symptomatic splenomegaly only				
Hydroxyurea	Inhibits ribonucleotide reductase and decrease DNA synthesis	500 to 1000 mg every other day	Clinical improvement in one-quarter to one-half of patients with MF along with ≥50 spleen volume reduction.	▪ Cytopenias ▪ Mucocutaneous ulcers ▪ Diarrhea ▪ Peripheral neuropathy ▪ Skin cancer ▪ Pulmonary toxicity
iii. Splenomegaly or other symptoms with anemia (platelets ≥50,000/microL)				
Ruxolitinib	JAK inhibitors	▪ 20 mg twice daily for a platelet count >200,000/ microL ▪ 15 mg twice daily for a platelet count between 100,000 and 200,000/ microL ▪ 10 mg twice daily for a platelet count between 75,000 and <100,000/ microL ▪ 5 mg twice daily for a platelet count between 50,000 and <75,000/microL	Ruxolitinib can relieve debilitating symptoms of PMF in up to one-half of patients, but it has not been shown to significantly prolong survival or reduce the risk of leukemic transformation in PMF.	▪ Full relapse of disease symptoms ▪ Fever ▪ Hypotenion ▪ Hypoxia ▪ Infections ▪ Ruxolitinib withdrawal syndrome
Fedratinib	JAK2-selective kinase inhibitor	400 mg once daily, with or without food; do not initiate fedratinib in patients with thiamine deficiency.	Reduced splenomegaly and symptoms in more than one-third of patients.	▪ Wernicke-like encephalopathy ▪ Hepatotoxicity ▪ Cardiovascular conditions
iv. Splenomegaly or other symptoms with anemia (<50,000 platelets/microL)				
Pacritinib	JAK2 and FMS-like tyrosine kinase 3 inhibitor	200 mg twice daily by mouth, with or without food.	In the phase 3 PERSIST-2 trial, pacritinib was more effective than best available therapy for spleen volume reduction and reducing symptom burden in patients with MF with thrombocytopenia.	▪ Hemorrhage ▪ Diarrhea ▪ Prolonged QT interval ▪ Thrombosis

Most MF patients experience anemia during the progression of the disease, resulting in the discontinuation of the ongoing treatment. These patients then need blood transfusions (7). However, the United States Food and Drug Administration (US-FDA) approved Momelotinib (MMB) on 15 September 2023 for treating intermediate or high-risk myelofibrosis in adults with anemia (26). This is a landmark step in managing MF as it is the first and only approved treatment for newly diagnosed and previously treated myelofibrosis patients who are experiencing anemia (27).

## Mechanism of action of momelotinib

**Figure 1:**
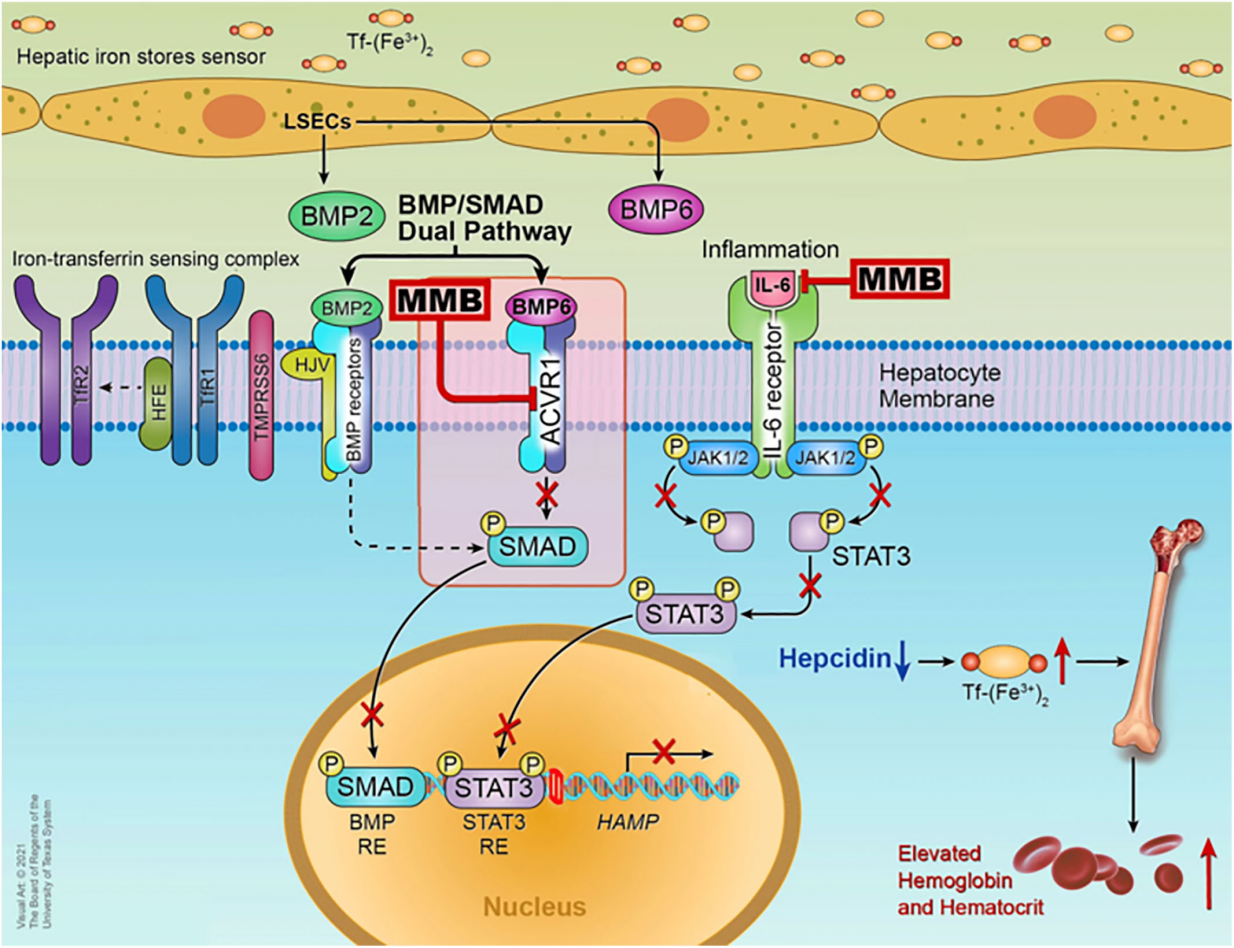
Mechanism of action of MMB ^©^ Chifotides HT, Bose P, Verstovsek S. CC BY 4.0 (29).

MMB is a small adenosine triphosphate (ATP)-a competitive molecule that effectively inhibits *JAK1* and *JAK2* in their typical forms and the mutant *JAK2V617F* variant (28). MMB and its primary metabolite in the human body, called M21, demonstrate a more substantial inhibitory effect on *JAK2* when compared to *JAK3* and tyrosine kinase 2 (TYK2) (17). The beneficial effects of MMB in addressing anemia and reducing the need for blood transfusions are associated with its ability to suppress the production of hepcidin mediated by Activin A receptor type I (ACVR1) also known as activin receptor-like kinase-2 (ALK2). ACVR1 plays a vital role in the production of blood cells and the development of anemia by influencing the BMP6/ACVR1/SMAD pathway, which regulates the expression of hepcidin, a key controller of iron balance in the body. In MF patients, hepcidin levels are elevated due to excessive activation of BMP6-stimulated ACVR1/ALK2 signaling and increased IL-6-driven inflammatory cytokine signaling. Suppressing liver hepcidin expression raises circulating iron and hemoglobin levels, thereby enhancing erythropoiesis in the bone marrow (29). Higher levels of hepcidin are linked to lower levels of iron in the blood, and when hepcidin is consistently high, it can lead to anemia due to insufficient iron. Anemia and the need for blood transfusions are linked to poor survival outcomes in MF patients (17). Research by Mora et al., which largely included patients who were not exposed to JAK inhibitors, found anemia correlated with higher prognostic risk categories, cytopenic phenotype and higher incidence of evolution into blast phase (BP) (30), while Palandri et al. study, primarily involving ruxolitinib-treated patients, showed that baseline or treatment-emergent anemia increased the risk of BP development and significantly worsened BP-free survival (31). Among the JAK inhibitors approved for treating MF MMB and pacritinib is often preferred for patients with low blood cell counts. Both MMB and pacritinib are potent inhibitors of ACVR1, effectively reducing hepcidin expression via the BMP6/ACVR1/SMAD pathway and restoring the balance of iron in the body, thereby aiding in the production of red blood cells although the benefits of pacritinib versus anemia are not yet well defined (32). The mechanism is well explained in the **Figure 1**.

Importantly, elevated hepcidin levels are significantly linked to shorter overall survival (OS) in patients with myelofibrosis (33). The potential therapeutic targeting of ACVR1 raises promising prospects for MMB in treating other myeloid neoplasms characterized by ineffective erythropoiesis, such as myelodysplastic syndromes involving ring sideroblasts or the Splicing Factor 3B Subunit 1A (SF3B1) mutation, particularly when these conditions are co-expressed with a *JAK2* mutation and thrombocytosis (34). The FDA approval of MMB is backed by the pivotal MOMENTUM trial (NCT04173494), and a subgroup of the patients experiencing anemia with MF symptoms participated in the SIMPLIFY-1 phase 3 trial (35). The primary outcome of these trials was to establish the efficacy and safety of MMB in MF patients.

## MOMENTUM trial

The MOMENTUM trial was a double-blind, active-controlled study that used a 2:1 randomized design to compare the efficacy of MMB against danazol in treating individuals with MF-related symptoms and anemia. This trial enrolled 195 patients who had previously been treated with JAK inhibitors. Before starting the trial, patients tapered off their existing JAK inhibitor treatment over more than a week and then underwent a non-treatment phase of at least two weeks. After completing a week-long baseline assessment period, participants were randomized using a biased coin minimization procedure to either the MMB group or the danazol group (33). This randomization aimed to minimize imbalances between the two groups based on factors such as Myelofibrosis Symptom Assessment Form (MFSAF; version 4.0) Total Symptom Score (TSS) (<22 vs ≥22), baseline palpable spleen length below the left costal margin (<12 cm vs ≥12 cm), baseline red blood cell or whole blood units transfused in the 8 weeks before randomization (0 units vs 1–4 units vs ≥5 units), and investigational sites (33). During the 24-week randomized treatment, patients in the MMB group received 200 mg orally once daily with danazol placebo, while the danazol group received 300 mg orally twice daily with MMB placebo. Both MMB and danazol doses were progressively reduced. MMB decreased by 50 mg increments, while danazol dropped by 200 mg initially and 100 mg subsequently, with minimum allowed doses of 50 mg for MMB and 200 mg for danazol (33). The trial’s primary objective was to assess the MFSAF TSS response rate at the end of the 24 weeks (33). Efficacy analyses followed the intention-to-treat principle, incorporating data from all randomly assigned patients, and the intention-to-treat and safety populations were identical. To control the overall type I error in the study, the evaluation of five key secondary endpoints was planned in hierarchical order. This assessment was to occur only if the primary endpoint demonstrated significance (two-sided p ≤ 0·05) in favor of MMB. The hypothesis test for transfusion independence rate at week 24 was focused on non-inferiority within the hierarchy, while superiority was tested within the hierarchy for all other endpoints (33). MMB demonstrated superiority over danazol, as evidenced by the mean TSS change from baseline at week 24 (−11·5 vs −3·9; least squares mean difference −6·2 [95% CI −10·0 to −2·4]; p=0·0014). Additionally, the rate of zero transfusions up to week 24 was higher in the MMB group (46 [35%; 95% CI 27–44] of 130) as compared to the danazol group (11 [17%; 9–28] of 65) (**Table 2**). The trial findings also revealed a significantly higher percentage of patients in the MMB group reporting a 50% or greater reduction in their TSS compared to the danazol group (32 [25%] of 130 vs. 6 [9%] of 65; proportion difference 16% [95% CI 6–26], p=0·0095) (33).

**Table 2:** Summary of MOMENTUM trial.

• Primary and key secondary efficacy endpoint analyses at week 24					
	Test Order	Criterion for significance	Momelotinib group (n=130)	Danazol group (n=65)	p value
TSS response rate	1	Superiority (p ≤ 0·05)	32 (25%)	6 (9%)	Two-sided 0·0095 (superior)
Transfusion independence rate	2	Non-inferiority	39 (30%)	13 (20%)	One-sided 0·0116 (non-inferior)
Splenic response rate (≥25% reduction)	3	Superiority (p ≤ 0·05)	51 (39%)	4 (6%)	Two-sided <0.0001 (superior)
Absolute TSS change from baseline	4	Superiority (p ≤ 0·05)	-11.5	-3.9	Two-sided 0·0014 (superior)
Splenic response rate (≥35% reduction)	5	Superiority (p ≤ 0·05)	29 (22%)	2 (3%)	Two-sided 0·0011 (superior)
Rate of zero transfusions to week 24	6	Superiority (p ≤ 0·05)	46 (35%)	11 (17%)	Two-sided 0·0012 (superior)
• Treatment-emergent adverse events observed in in either treatment group					
	Momelotinib group (n=130)		Danazol group (n=65)		
	Any grade	Grade ≥ 3	Any grade	Grade ≥ 3	
1. Non-haematological abnormalities					
Diarrhea	29 (22%)	0	6 (9%)	1 (2%)	
Nausea	21 (16%)	3 (2%)	6 (9%)	2 (3%)	
Asthenia	17 (13%)	1 (1%)	6 (9%)	1 (2%)	
Pruritus	14 (11%)	2 (2%)	7 (11%)	0	
Weight ↓	14 (11%)	0	4 (6%)	0	
Blood Creatinine ↑	10 (8%)	1 (1%)	10 (15%)	2 (3%)	
Dyspnea	10 (8%)	3 (2%)	9 (14%)	1 (2%)	
Peripheral Oedema	10 (8%)	2 (2%)	9 (14%)	0	
Fatigue	8 (6%)	1 (1%)	7 (11%)	2 (3%)	
Acute Kidney Injury	6 (5%)	4 (3%)	8 (12%)	6 (9%)	
Peripheral Neuropathy	5 (4%)	0	1 (2%)	0	
2. Hematologic abnormalities					
Anemia	129 (99%)	79 (61%)	65 (100%)	49 (75%)	
Thrombocytopenia	99 (76%)	36 (28%)	40 (40%)	17 (26%)	
Neutropenia	38 (29%)	16 (12%)	17 (26%)	6 (9%)	

The trial also indicated that a significant number of patients initially required blood transfusions. At the beginning of the study, 20% of the patients in the MMB group (26 out of 130) and 17% in the danazol group (11 out of 65) did not need any red blood cell (RBC) transfusions for 28 days. As the trial progressed, the effectiveness of the MMB treatment in reducing the need for RBC transfusions became evident (36). During the treatment phase, 35% of the patients in the MMB group (46 out of 130) did not require any transfusions, compared to only 17% in the danazol group (11 out of 65). Notably, among those who did not need transfusions at the start, a higher percentage of patients treated with MMB (92%) continued to require no transfusions during the treatment phase, compared to 64% in the danazol group. Moreover, there was a noticeable decrease in the average number of RBC units needed per patient every 28 days (36). In the MMB group, this number dropped by 0.86 units (SD=1.748), while in the danazol group, the decrease was 0.28 units (SD=1.584). The trial also categorized changes in transfusion needs into different levels. In the MMB group, a substantial 85% of patients either maintained (19.2%) or improved (65.4%) their transfusion requirements compared to the baseline. In contrast, in the danazol group, 63% of patients either maintained (10.8%) or improved (52.3%) their transfusion requirements (36).

A *post hoc* time-dependent analysis of the MOMENTUM trial also investigated the prognostic influence of RBC transfusion status over time and other covariates on OS. RBC transfusion status was the consistent prognostic variable while only RBC transfusion status was significantly associated with survival. The study found a compelling correlation between transfusion status and overall survival. Specifically, the data revealed that patients who were non transfusion-independent had a five times higher risk of all-cause mortality compared to those who were transfusion-independent (HR, 5.18; 95% CI, 1.86–14.47; P=.0017) (37). Hence, MMB group had greater number of patients with lower risk of all-cause mortality. Another study described the descriptive responder, longitudinal responder, and time-to-event analyses that supported the primary endpoint of the MOMENTUM trial. The results indicate that MMB led to more consistent and significant symptom relief compared to danazol. Patients treated with MMB experienced early and ongoing reductions in symptoms such as night sweats, abdominal discomfort, rib pain, and bone pain, with notable improvements starting as early as day 29. Additionally, MMB was more effective in reducing both disease-related and cancer-related fatigue and enhancing physical functioning. These findings collectively suggest that MMB offers a rapid, progressive, and sustained benefit, enhancing the quality of life for patients with MF (38).

The most common treatment-emergent adverse effects are also summarized in **Table 2**. The most commonly reported hematological abnormalities based on laboratory values for both MMB and danazol were treatment-related anemia, which occurred in 79 (61%) out of 130 patients in the MMB group and 49 (75%) out of 65 patients in the danazol group, as well as thrombocytopenia, affecting 36 (28%) out of 130 patients in the MMB group and 17 (26%) out of 65 patients in the danazol group. Regarding non-hematological grade 3 or higher treatment-emergent AEs, MMB and danazol had different profiles (33). MMB resulted in acute kidney injury in four patients (3%) out of 130 and pneumonia in three patients (2%) out of 130. In comparison, danazol led to acute kidney injury in six patients (9%) out of 65 and pneumonia in six patients (9%) out of 65. Additionally, peripheral neuropathy (grade ≤2) occurred in five patients (4%) receiving MMB and one patient (2%) receiving danazol, with no study drug discontinuations reported (33).

The current treatment of myelofibrosis is constrained by the myelosuppressive effects of approved JAK inhibitors. However, MMB presents dual JAK1/2 and ACVR1 inhibition, which provides enhanced therapeutic efficacy in MF. The study demonstrates rapid and sustained improvements in hemoglobin concentrations, non-inferior transfusion-independent rates, a superior rate of zero transfusions, and fewer transfusions compared to danazol (33). MMB’s unique ability to maintain higher doses due to reduced myelosuppressive activity is highlighted. Safety profiles and efficacy in subgroups with thrombocytopenia are consistent, supporting its use in patients with low platelet counts. Despite the challenges posed by the COVID-19 pandemic, the MOMENTUM study, completed within the planned timeframe, shows a trend towards improved OS for the MMB group, with ongoing patient follow-up for long-term survival analyses (33).

The MOMENTUM study faced limitations with its week 24 crossover design, preventing a direct, prolonged survival comparison between treatment groups. There was a potential for bias as patients and investigators might have anticipated treatment assignments, but danazol-treated individuals exhibited advantages in key efficacy measures, mitigating this concern. Although the possibility of early study discontinuation was recognized, a majority of patients with advanced myelofibrosis in both groups completed the randomized treatment phase. The observed higher rate of transfusion independence in MMB-randomized splenic responders at week 24 is confounded by the disproportionate assessment availability, as early discontinuations were deemed non-responders (33).

## SIMPLIFY-1 and SIMPLIFY-2 trial

The SIMPLIFY-1 trial was a phase 3 study that included 432 patients with primary myelofibrosis (PMF), post-polycythemia vera, or post-essential thrombocythemia. These patients were JAK inhibitor-naïve and classified as intermediate- or high-risk. The study aimed to compare the efficacy of two treatments, MMB and ruxolitinib, over a 24-week period. Participants were randomly assigned to receive either MMB or ruxolitinib, with the MMB group taking 200 mg once daily and the ruxolitinib group taking 5–20 mg twice daily. In total, 430 subjects received at least one dose of the study treatment, and at week 24, 91.2% (197 out of 216) of ruxolitinib-randomized patients transitioned to MMB treatment (39). Out of these 432 patients, a subgroup of 181 had anemia and MF symptoms at the time of entry (35).

The main goal of the study was to observe if there was a reduction of at least 35% in spleen size after 24 weeks of treatment. Secondary end points were rates of symptom response and effects on RBC transfusion requirements (39). At the 24-week mark, approximately the same percentage of patients in both the momelotinib and ruxolitinib groups saw a ≥ 35% reduction in spleen volume, with 26.5% in the momelotinib group and 29% in the ruxolitinib group, demonstrating that momelotinib was not inferior to ruxolitinib (noninferior; P = .011). However, when it came to reducing the total symptom score by 50% or more, 28.4% of patients on momelotinib and 42.2% on ruxolitinib achieved this, meaning momelotinib did not meet the noninferiority standard for symptom reduction (P = .98). Nonetheless, momelotinib showed better outcomes in terms of reducing the need for transfusions, achieving transfusion independence, and reducing transfusion dependence, all with statistically significant improvements (nominal P ≤.019) (39).

RBC transfusion needs in patients treated with MMB versus ruxolitinib was also evaluated in a study (36). Initially, 70% (150 of 213) of the MMB group and 76% (163 of 216) of the ruxolitinib group did not require transfusions. Over 24 weeks, 95% (142 of 150) of these patients in the MMB group remained transfusion-free, compared to 57% (93 of 163) in the ruxolitinib group. The MMB group experienced a slight reduction in RBC units needed per 28 days (average decrease of 0.10 units), while the ruxolitinib group saw an increase (average rise of 0.39 units). Additionally, 87% of MMB patients maintained or improved (144 [67.6%]) or improved (41 [19.2%]) their transfusion status, compared to 54% (maintained, 94 [43.5%]; improved, 23 [10.6%]) in the ruxolitinib group (36). Another study indicated that age, platelet count, and initial spleen volume were significantly linked to OS in SIMPLIFY-1 trial. When adjusting for differences in prognostic factors, effect modifiers, and changes in transfusion status over time, the findings from SIMPLIFY-1 showed a strong and significant correlation between transfusion status and overall survival (HR, 3.32; 95% CI, 2.31–4.78; P<.0001). This indicates that patients who were not transfusion-independent had a more than threefold increased risk of all-cause mortality compared to those who were transfusion-independent (37).

The MMB and ruxolitinib groups both showed similar overall improvements in symptoms, with a TSS of 17.4 in the MMB group and 16.4 in the ruxolitinib group, with a difference of less than 1.5 points (40). The survival outcomes did not significantly differ between JAK inhibitor-naïve patients randomly assigned to MMB and those initially given ruxolitinib followed by MMB in the SIMPLIFY-1 study, as indicated by the similar survival distributions (Overall Survival Hazard Ratio [OS HR] = 1.02 [95% CI: 0.73, 1.43]; Leukemia-Free Survival Hazard Ratio [LFS HR] = 1.08 [0.78, 1.50]) (41).

In this trial, 35.5% of patients receiving MMB experienced Grade 3 or higher AEs, compared to 43.5% of those on ruxolitib (39). The most frequently reported grade 3 or 4 AEs with MMB included thrombocytopenia (7.0%), anemia (5.6%), diarrhea, hypertension, and neutropenia (2.8% each). For ruxolitinib, the most commonly reported grade 3 or 4 AEs were anemia (23.1%), neutropenia and thrombocytopenia (4.6% each), and hypertension (4.2%). Moreover, peripheral neuropathy (all grade ≤ 2) occurred in 22 patients (10.3%) receiving MMB and 10 patients (4.6%) receiving ruxolitinib (all grade ≤ 3) (39). Serious AEs were noted in 22.9% of MMB-treated patients and 18.1% of ruxolitinib-treated patients (**Table 3**). AEs leading to discontinuation of the study drugs occurred in 13.1% of patients taking MMB and 5.6% of patients taking ruxolitinib. Additionally, AEs resulting in dose reduction or temporary interruption of the study drugs were reported by 17.8% of patients receiving MMB and 36.6% of patients receiving Ruxolitinib (11).

**Table 3:** Summary of SIMPLIFY-1 trial.

• Primary and key secondary endpoint analyses at week 24					
	Test Order	Criterion for significance	Momelotinib group (n=215)	Ruxolitinib group (n=217)	P value
Spleen response rate (≥ 35% in spleen volume from baseline)	1	Non-inferiority was met (lower bound of the two-sided 95% CI > 0)	57 of 215 (26.5%)	63 of 217 (29%)	0.011
TSS response rate (≥ 50% reduction from baseline)	2	Non-inferiority was not met.	60 of 211 (28.4%)	89 of 211 (42.2%)	0.98
RBC-transfusion independence rate (proportion of patients who were transfusion-independent at week 24)	3	–	143 of 215 (66.5%)	107 of 217 (49.3%)	<.001
RBC-transfusion dependence rate	4	–	65 of 215 (30.2%)	87 of 217 (40.1%)	0.019
Rate of RBC transfusion (average number of RBC units per subject-month during treatment)	5	–	0 units/mo	0.4 units/mo	*P* <.001
• Treatment-Emergent Adverse Events Occurring in ≥ 10% of Either Treatment Group					
	Momelotinib (n=214)			Ruxolitinib (n=216)	
Thrombocytopenia	40 (18.7%)			63 (29.2%)	
Diarrhea	38 (17.8%)			43 (19.9%)	
Headache	37 (17.3%)			43 (19.9%)	
Dizziness	34 (15.9%)			25 (11.6%)	
Nausea	34 (5.9%)			8 (3.7%)	
Fatigue	31 (14.5%)			26 (12%)	
Anemia	29 (13.6%)			82 (38%)	
Abdominal Pain	22 (10.3%)			24 (11.1%)	
Peripheral Neuropathy	22 (10.3%)			10 (4.6%)	

The SIMPLIFY-2 trial included the MF patients already treated with JAK inhibitors. It was a phase 3 study with a 2:1 randomization, conducted internationally in an open-label manner. The study aimed to establish the superiority of MMB over the best available therapy in individuals with PMF, post-polycythemia vera myelofibrosis, or post-essential thrombocythemia myelofibrosis who had previously undergone treatment with ruxolitinib and experienced hematologic toxicity (n=156) (40). All 156 subjects received the designated study treatment, and among the best available therapy/ruxolitinib randomized patients, 76.9% (40 out of 52) switched to MMB treatment at week 24.

The primary endpoint for both trials was the reduction of spleen volume by at least 35% from baseline at the 24-week mark. Secondary endpoints included response rates for TSS and red blood cell transfusion independence at 24 weeks, along with OS and LFS. The results of this trial indicated improvement in TSS in the MMB group, which was in line with what was seen in the SIMPLIFY-1 trial (40). In SIMPLIFY-2, patients previously exposed to ruxolitinib showed a two-year OS of 65.8% and LFS of 64.2% with MMB, compared to 61.2% and 59.7% with the best available therapy followed by MMB (OS HR = 0.98 [0.59, 1.62]; LFS HR = 0.97 [0.59, 1.60]). The presence of baseline transfusion independence was linked to improved survival in both SIMPLIFY-1 (HR = 0.474, p = 0.0001) and SIMPLIFY-2 (HR = 0.226, p = 0.0005) studies (41). The likelihood of symptom improvement was higher in the MMB group (42). The results of SIMPLIFY-1 and SIMPLIFY-2 demonstrated that MMB effectively improves clinically related symptoms of MF patients irrespective of whether they had previously received any treatment. The results and the major AEs of both SIMPLIFY trials are summarized in the **Tables 3**, **4** (35).

**Table 4:** Summary of SIMPLIFY-2 trial.

• Primary and key secondary endpoint analyses at week 24						
	Test Order	Momelotinib group (n=104)		BAT group (n=54)		P value
Spleen response rate (≥ 35% in spleen volume from baseline)	1	7 of 104 (7%)		3 of 52 (6%)		0.90
TSS response rate (≥ 50% reduction from baseline)	2	27 of 103 (26%)		3 of 51 (6%)		0.0006
RBC-transfusion independence rate (proportion of patients who were transfusion-independent at week 24)	3	45 of 104 (43%)		11 of 52 (22%)		0.0012
RBC-transfusion dependence rate	4	52 of 104 (50%)		33 of 52 (64%)		0.10
Rate of RBC transfusion (average number of RBC units per subject-month during treatment)	5	0.5 units/mo		1.2 units/mo		0.39
• Treatment-Emergent Adverse Events Occurring in ≥ 10% of Either Treatment Group						
	Momelotinib (n=104)			BAT (n=54)		
	Grade 1/2	Grade 3	Grade 4	Grade 1/2	Grade 3	Grade 4
Nausea	18 (17%)	2 (2%)	0	4 (8%)	1 (2%)	0
Asthenia	15 (14%)	5 (5%)	0	10 (19%)	1 (2%)	0
Cough	18 (17%)	0	0	6 (12%)	0	0
Abdominal Pain	15 (14%)	1 (1%)	0	5 (10%)	3 (6%)	0
Anaemia	4 (4%)	12 (12%)	2 (2%)	1 (2%)	7 (14%)	0
Fatigue	15 (14%)	1 (1%)	0	9 (17%)	1 (2%)	0
Headache	15 (14%)	1 (1%)	0	2 (4%)	1 (2%)	0
Dizziness	16 (15%)	0	0	4 (8%)	0	0
Pyrexia	13 (13%)	2 (2%)	0	4 (8%)	0	0
Dyspnoea	11 (11%)	2 (2%)	0	6 (12%)	1 (2%)	0
Pruritus	12 (12%)	1 (1%)	0	4 (8%)	0	0
Thrombocytopenia	6 (6%)	2 (2%)	5 (5%)	3 (6%)	2 (4%)	1 (2%)
Constipation	12 (12%)	0	0	2 (4%)	0	0
Urinary Tract Infection	9 (9%)	2 (2%)	0	4 (8%)	0	0
Peripheral Edema	10 (10%)	0	0	6 (12%)	0	0
Epistaxis	8 (8%)	0	0	6 (12%)	0	0
Bone pain	2 (2%)	0	0	6 (12%)	0	0
Peripheral Neuropathy	10 (10%)	1 (1%)	0	0	0	0

*Data are n (%). BAT, best available therapy.

The mature survival data from the phase 3 SIMPLIFY trials reveal that extended treatment with MMB demonstrates excellent OS and LFS in both JAK inhibitor-naïve and previously ruxolitinib-treated patients. The non-inferiority study, SIMPLIFY-1, shows nearly identical OS and LFS outcomes for patients initially randomized to MMB or ruxolitinib. While cross-study comparisons are challenging, the 5-year survival probability is approximately 55% in both arms of SIMPLIFY-1 (41). In SIMPLIFY-2, which involved ruxolitinib-exposed patients, extended MMB treatment exhibits a median OS of around 3 years and a 2-year survival rate of 61–66%, demonstrating promising survival post-ruxolitinib. MMB’s unique inhibition of JAK1, JAK2, and ACVR1/ALK2, a key player in iron homeostasis, leads to decreased hepcidin and increased serum iron availability, contributing to considerable anemia benefits. The study suggests that MMB, with its marked anemia and transfusion independence benefits, maybe a preferred treatment choice for specific subsets of myelofibrosis patients, potentially influencing future treatment decisions (41).

The week 24 crossover design of the SIMPLIFY trials introduces inherent limitations that may impact the OS findings in both SIMPLIFY-1 and SIMPLIFY-2 studies. Due to the crossover, the direct comparability of OS data between the MMB and control arms is compromised, making it challenging to accurately estimate the treatment effect of MMB. Although the studies aimed to provide 24-week comparative data, the survival outcomes are more descriptive for extended MMB treatment, as most control arm patients switched to MMB early in comparison to the median survival follow-up exceeding three years (41). Additionally, the SIMPLIFY-2 design lacked a washout period for prior JAK inhibitor therapy, potentially influencing the specificity of MMB effects and contributing to the non-significant association between week 24 clinical endpoints and OS in this study. Future research is necessary to identify factors beyond baseline hemoglobin and transfusion requirements that could predict which MMB-treated patients are likely to respond positively or negatively to transfusion independence (41).

## Conclusion

Currently, allogeneic stem cell transplantation is the sole viable treatment for achieving long-term survival in MF, with a notable rise in the number of such transplants in recent years (43). For patients who are ineligible for transplantation, effective palliative care involves addressing key quality-of-life concerns, namely anemia, splenomegaly, and constitutional symptoms. MMB is considered a promising treatment for managing the adults with intermediate- or high-risk MF with anemia, particularly because it can help alleviate anemia and potentially enhance outcomes for patients with blood cancers. While this development is encouraging for those suffering from anemia related to MF, there are important considerations to keep in mind. Firstly, as a JAK inhibitor, MMB can have side effects such as immune suppression, making patients more susceptible to infections. Therefore, careful monitoring for opportunistic infections is essential. Additionally, patients need to be informed about the risk of drug-induced peripheral neuropathy and should be regularly assessed for this condition. On a positive note, using momelotinib in combination with other agents that stimulate red blood cell production or reduce disease burden holds promise for improving symptom relief in MF. However, it is important to note that, similar to other JAK inhibitors, momelotinib does not appear to reverse the structural or molecular characteristics of MF, nor does it seem to alter the disease’s progression. Though the statistical and clinical effectiveness of MMB has been established, the cost-effectiveness of MMB still needs to be established. Further studies are also required to assess the impact of this drug on other myeloid neoplasms associated with anemia such as myelodysplastic syndromes with ring sideroblasts or *SF3B1* mutation, especially those with co-expression of a *JAK2* mutation and thrombocytosis. We are optimistic that these recommendations could open a new paradigm for treating patients with coexisting anemia and myeloid neoplasms.

## Author contributions

MF: Conceptualization, Data curation, Investigation, Methodology, Project administration, Supervision, Writing – original draft, Writing – review & editing. MO: Data curation, Methodology, Writing – review & editing.
